# Pharmacogenetics of CYP2C19 genetic polymorphism on clopidogrel response in patients with ischemic stroke from Saudi Arabia 

**DOI:** 10.17712/nsj.2017.1.20160303

**Published:** 2017-01

**Authors:** Adel A. Alhazzani, Murali Munisamy, Gauthaman Karunakaran

**Affiliations:** *From the Department of Neurology (Alhazzani), College of Medicine, and from College of Pharmacy (Munisamy, Karunakaran), King Khalid University, Abha, Kingdom of Saudi Arabia*

## Abstract

**Objective:**

To elucidate the degree of genetic polymorphisms CYP2C19 (CYP2C19*2, CYP2C19*3) of key drug metabolizing enzymes on the antiplatelet effect of clopidogrel response in patients with acute ischemic stroke from Saudi Arabia.

**Methods:**

A case-control study carried out at Neurology Clinics at Asser Central Hospital, Abha, Kingdom of Saudi Arabia from October 2015 to January 2016 and included 25 stroke patients responding to clopidogrel therapy and 25 stroke patients non responding to clopidogrel monotherapy. After obtaining their informed consent, the blood samples were collected and genotyped for CYP2C19 polymorphisms by the polymerase chain reaction-restriction fragment length polymorphisms (PCR-RFLP Method). Allele frequencies were derived from genotypic data and platelet aggregation was measured using multiple electrode aggregometry on the multiplate analyser. Chi Square tests, p-values, odds ratio (OR) and corresponding confidence intervals were calculated for each polymorphism.

**Results:**

The CYP2C19*2 (681G>A) and CYP2C19*3 (636 G>A) polymorphism were seen to be in Hardy–Weinberg equilibrium and showed significant allelic and genotypic association between responders and non-responders to clopidogrel (*p*<0.01). The CYP2C19*2: allelic chi-square=21.49, *p*=0.000036, OR=5.52 (2.42-12.83); Genotypic Chi-square=10.27, *p*=0.001, OR=7.88 (1.78-9.73). The CYP2C19*3: Allelic chi-square=11.66, *p*=0.0006, OR=3.45 (1.57-7.70); genotypic chi-square=4.37, *p*=0.036, OR=3.69 (0.90-5.81). The variant allele (homozygous and homozygous Mutant) showed significant influence on platelet inhibition and the antiplatelet effect of clopidogrel in ischemic stroke.

**Conclusion:**

Our findings provide certain evidence on the genetic effect of CYP2C19 on clopidogrel responsiveness in stroke patients from Saudi Arabia.

Stroke is a major cause of adult neurological disability and mortality. It is the second most common cause of death worldwide and affects almost 200 per 100,000 people every year.^1^ In Arab nations, the annual stroke incidence varies between 27.5 to 63 per 100,000 people, and the prevalence is 42 and 68 per 100,000 people.^2^ In Saudi Arabia, the prevalence of stroke was estimated at 43.8 per 100,000 per year in Riyadh and 40 per 100,000 in the Eastern Province, with a male to female predominance of 2.2:1. Increasing public awareness of stroke risk factors and warning signs has been widely recognized as a crucial factor influencing stroke prevention, prehospital care, and therapeutic outcomes.

The primary mechanisms for a stroke are either blockage of blood flow, causing ischemia, or rupture of a blood vessel, resulting in hemorrhage into the surrounding tissue.^3^ Clinically, it is divided into subtypes based upon the responsible mechanism, namely hemorrhagic versus ischemic. Approximately 80-85% of stroke cases are ischemic, whereas 15-20% are hemorrhagic. Stroke is a multifactor disease that is thought to be caused by a combination of environmental and genetic factors, with an ever-increasing rate of incidence. To improve diagnosis and treatment strategies, as well as to reduce the related public health burden, it could be helpful to identify the extremely complex genetic determinants of stroke (polygenic, multiple genes).

Until recently, the main technique used to look for genes predisposing to common stroke was the candidate gene method. Using this method, genetic variants, usually single-nucleotide polymorphisms (SNPs) are identified in a ‘candidate’ gene that is thought to be involved in stroke risk. Pharmacogenetics has long held the promise of individualizing pharmacological therapy using genetic biomarkers. Pharmacogenomics has a role in choosing therapies and identifying the optimal dose according to the individual’s genotype pattern. Clopidogrel has an ability to minimize the risk of recurrent ischemic stroke like aspirin.^4^ Clopidogrel is widely used for prevention of secondary stroke. Approximately 5-30% of clopidogrel-treated patients exhibit low or no reactivity to clopidogrel, which is referred to as clopidogrel resistance. Clopidogrel resistance is a term used to describe a suboptimal response to the platelet inhibitory effects desired from treatment with clopidogrel. Poor response to clopidogrel may be present in up to 21% of patients treated, and these patients may be at up to 12 times greater risk for recurrent ischemic events.^5^

Clopidogrel requires transformation into an active metabolite by cytochrome P450 (CYP) for its anti-platelet effect. Different CYP isoenzymes are responsible for clopidogrel activation, and among these, CYP2C19 has been found to play a key role. Clopidogrel is a prodrug that is converted to an activate thiol metabolite by several cytochrome P450 (CYP) micro enzymes (for example, CYP2C19, 3A4, 3A5, 2C9, among others) in a 2-step process accompanied by the formation of an intermediate metabolite. Among the enzymes mediating this conversion, CYP2C19 contributes to 44.9% of the conversion of clopidogrel to 2-oxoclopidogrel, and around 20% to the formation of the active thiol metabolite from 2-oxo-clopidogrel. Thus, CYP2C19 plays a dominant role in both of the clopidogrel activation steps.^6^

Platelet testing may provide an objective measure of compliance. However, for patients who have been treated with an irreversible antiplatelet drug during a hospital stay, it will not be possible to identify clopidogrel responders using platelet testing. Genotyping, in this circumstance, is a logical option. Genotyping prior to drug administration may be of particular importance for drugs like clopidogrel, which is often started in the acute setting with the need for a rapid and effective antiplatelet effect. Although phenotyping, using platelet function testing, provides a more integrated assessment of drug response, testing can only be performed after a drug is administered. In addition, genomics can provide information on many more dimensions of health than just the response to one drug. Thus, combining genotyping and phenotyping may be more effective in predicting clinical outcomes than either alone. The aim of our study was, therefore, to assess the influence of CYP2C19 genetic polymorphisms on the response to clopidogrel in ischemic stroke in Saudi Arabian population.

## Methods

### Study subjects

This single-center, prospective observational cohort, case control study included 50 patients of Aseer region descent suffering from stroke patients. Patients with 75 mg of maintenance dose of clopidogrel therapy were attending Neurology Clinics at Aseer Central Hospital, Abha, Kingdom of Saudi Arabia, between October 2015 and January 2016 were included. The study protocol was approved by the Ethics Committee and all studies were performed with full and informed consent of the patients. All patients were diagnosed as acute cerebral ischemic stroke, which was diagnosed by a neurologist according to the diagnostic criteria determined by the guidelines for the primary prevention of stroke: a guideline for healthcare professionals from the American Heart Association/ American Stroke Association^7^ and confirmed by CT scan or conventional MRI of the brain.

### Sample size calculation

Sample size was calculated taking parameters of the study published by Sukasem et al^8^ in which the prevalence of responders was 71%, and prevalence of non-responders was 29% in the study population. Taking 80% power, 0.05 alpha (2 sided), one sample per case, we obtained the estimated sample size of 50 (25 in each group). Sample size was calculated using STATA software version 13. Group one comprised 25 stroke patients that were non- responders to clopidogrel therapy. Group 2 comprised 25 stroke patients that were responders to clopidogrel therapy.

Subjects were included in the study they had received a 75 mg maintenance dose of clopidogrel at least 6 hours prior to blood sampling. Subjects were excluded if their platelet counts were lower than 100,000 per mm^3^, or had cerebral venous thrombosis and neurodegenerative disease. In addition, patients were excluded when they took proton pump inhibitor, tricyclic antidepressants, antiepileptic’s and antipsychotics, warfarin, or glycoprotein IIb/IIIa antagonist. Patients with a history of drug or alcohol abuse, bleeding disorder, myelodysplastic or myeloproliferative disorders, chronic liver disease or any contraindication against clopidogrel were all excluded. Informed consent was obtained from all subjects who participated in the study.

### Molecular analysis, blood sample collection, and genotyping

Five ml blood sample, (2 ml in EDTA coated vials for DNA isolation, and 3 ml in plain tubes for serum separation for performing platelet aggregation study) were collected by single time venipuncture. Each sample was assigned a consecutive sample number and used for DNA isolation and genotyping. The patient’s demographic and risk factor information was collected in the standardized data collection form at the time of blood sample collection in which available clinical and imaging data were collected. Samples were used for genomic DNA isolation from white blood cells by using the phenol chloroform isolation method and extracted genomic DNA was dissolved in 200-600 ml TE buffer depending upon the concentration of DNA and stored at -20°C. DNA was isolated on a weekly basis for the isolation of good quality DNA. Its quality was checked first in 0.8% agarose gels. Quality of DNA in per µl was checked in Nano drop spectrophotometer. The purity of the DNA sample was ascertained by calculating a 260/280 ratio. A ratio between 1.5-1.8 was considered as acceptable for polymerase chain reaction amplification. Polymerase chain reaction–restriction fragment length polymorphisms (PCR–RELPs) for the CYP2C19 loss-of-function (LOF) alleles on 2 polymorphic positions, *2 (681G4A, rs4244285) and *3 (636G4A, rs4986893), were performed as previously described method.^9^,^10^ The primers of CYP2C19 *2 and *3 were designed, and fast-digest restriction enzymes SmaI and BamHI for CYP2C19 *2 and *3 were used.

### Platelet aggregation assays

Two ml of peripheral venous blood samples were obtained from subjects in a catheterization laboratory prior to the next dose of clopidogrel. Platelet aggregation was measured using Multiple Electrode Aggregometry (MEA) on the Multiplate analyser (Dynabyte, Munich Germany). Blood was placed in 4.5 ml plastic tubes containing hirudin with a final concentration of 25 mg/ml. The final concentration of Adenosine diphosphate (ADP) (6.5 mM) -induced platelet aggregation was assessed as previously reported.^11^ Platelet aggregation measured with MEA was quantified as area under the curve (AUC=AU6 min) of aggregation unit (AU). A 10 AU 6 min corresponds to one unit (U). The cut off point for this clopidogrel resistance was found to be 50 U as previously reported.^12^

### Statistical analysis

Analysis was performed using the Statistical Package for the Social Sciences version 19.0 (IBM SPSS Statistics for Windows, Armonk, NY, USA). Continuous variables were expressed as mean±standard deviation (SD) and categorical variables were reported as counts and percentages. Comparisons of the genotypic or allelic frequencies between groups were performed using the chi-squares test. Odd’s ratios were calculated with a 95% confidence interval limit from 2×2 contingency tables. A *p*-value <0.05 was considered significant. Deviations from the Hardy–Weinberg equilibrium were calculated. A value of *p*<0.05 (2-sided) was considered statistically significant. The association of ADP induced platelet aggregation level by CYP2C19*2,*3 polymorphisms in clopidogrel treated patients with ischemic stroke with genotypes was determined by Man Whitney U test, and *p*<0.05 was considered to be statistically significant.

## Results

**Figure 1:**
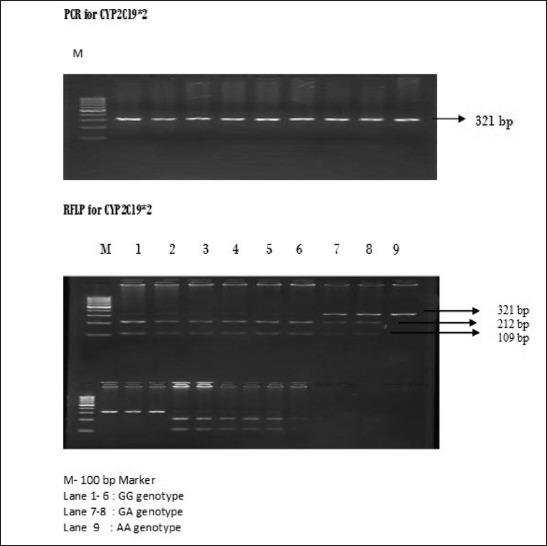
- Polymerase chain reaction and RFLP pattern of CYP2C19*2 (681 G>A) Polymorphism. RFLP - restriction fragment length polymorphism, PCR - Polymerase chain reaction

**Figure 2:**
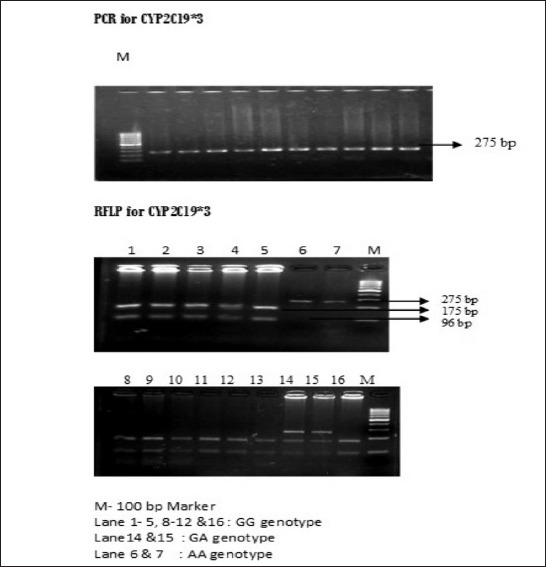
- Polymerase chain reaction and RFLP Pattern of (636 G>A) polymorphism. RFLP - restriction fragment length polymorphism, PCR - Polymerase chain reaction

Baseline clinical characteristics of the cerebral ischemic stroke group are shown in **Table 1**. Fifty patients with ischemic stroke (25 responders to clopidogrel, 25 non responders to clopidogrel monotherapy) cases were recruited. Their mean age of the responders group was 54.2 and the standard deviation was ±9.4. The mean age of the non responders group was 57.8 and the standard deviation was ±11.9. The proportions of hypertension, and diabetes were higher in the non-responders group. Based on the results from platelet function test using MEA, the ischemic stroke patients were categorized into responders and non-responders to clopidogrel. Among 50 patients included in this study, 25 patients were classified as non-responders and 25 patients as responders. Polymerase chain reaction-restriction fragment length polymorphism was used to examine CYP2C19*2 (681G>A) and CYP2C19*3 (636 G>A) genotype distributions in ischemic stroke patients. The gel photographs of CYP2C19 SNPs are represented in **Figure 1 and Figure 2**. These polymorphisms were polymorphic in our study population and were seen to be under the Hardy Weinberg equilibrium (cut off *p*<0.05). The genotype and allele frequencies of these variants for responders and non responders to clopidogrel are shown in **Table 2 and Table 3**.

**Table 1:** Baseline characteristics of stroke patients.

Demographic characteristics	Clopidogrel responders (n=25)	Clopidogrel non -responders (n=25)
Mean age±SD (years)	54.2±9.4	57.8±11.9
Gender (male: female), n (%)	18:7	19:6
Hypertension, n (%)	11 (44)	16 (64)
Diabetes, n (%)	13 (52)	17 (68)
Clopidogrel dose	75 mg	75 mg
Platelet count (x10^5^/mm^3^)	2.27±0.72	2.83±0.84

**Table 2:** Allelic frequency of CYP2C19 polymorphism among stroke patients.

Genes	Polymorphism	Allelic frequency of clopidogrel non responders n=25	Allelic frequency of clopidogrel responders n=25	Allelic chi-square value	*P*-value	Odds ratio and 95% CI
CYP2C19*2	681 G>A	G=0.62 A=0.38	G=0.90 A=0.10	Non responders vs responders: 21.49	0.00036	5.52 (2.42-12.83)*
CYP2C19*3	636 G>A	G=0.68 A=0.32	G=0.88 A=0.12	Non responders vs responders:11.66	0.0006	3.45 (1.57-7.70)*

vs - versus,

* significant

**Table 3:** Genotypic Frequency of CYP2C19 polymorphism among stroke patients.

Genes	Polymorphism	Genotypic frequency of clopidogrel non responders n=25	Genotypic frequency of clopidogrel responders n=25	Allelic chi-square value	*P*-value	Odds ratio and 95% CI
CYP2C19*2	681 G>A	GG=10 GA+AA=15	GG=21 GA+AA=4	Non responders Vs responders=10.27	0.001	7.88 (1.78-9.73)*
CYP2C19*3	636 G>A	GG=13 GA+AA=12	GG=20 GA+AA=5	Non responders Vs responders=4.37	0.036	3.69 (0.90-5.81)*

Vs - versus, CI - confidence interval,

* significant

For clopidogrel drug, the allelic and genotypic frequency for CYP2C9*2, CYP2C9*3 showed significant association between responders and non-responders group. The CYP2C19*2: allelic chi-square=21.49, *p*=0.000036, OR=5.52 (2.42-12.83); genotypic chi-square=10.27, *p*=0.001, OR=7.88 (1.78-9.73). The CYP2C19*3: allelic chi-square: 11.66, *p*=0.0006, OR=3.45 (1.57-7.70); genotypic chi-square=4.37, *p*=0.036, OR=3.69 (0.90-5.81). The ADP-induced platelet aggregation values across CYP2C19*2 and *3 genotypes are shown in **Table 4**. The ADP induced platelet aggregation (U) level for CYP2C19*2, CYP2C19*3 polymorphism for variant allele (heterozygous and homozygous mutant) was found to be higher (63.2±33.18, 58.29±28.18), compared to the wild type allele (37.1±23.18, 43.1±27.18). Adenosine diphosphate induced-platelet aggregation for CYP2C19*2, CYP2C19*3 variant genotypes were significantly different when compared with wild type genotype (*p*= 0.004).

**Table 4:** ADP induced platelet aggregation level by CYP2C19 polymorphisms in clopidogrel treated patients with ischemic stroke.

Gene	Polymorphism	Genotypes	N=50 (non responders + responders group)	Platelet aggregation level
CYP2C19*2	681 G>A	GG (*1/*1) GA+AA (*1/*2,*2/*2)	31 19	37.1±23.18 63.2±33.18
CYP2C19*3	636 G>A	GG (*1/*1) GA+AA (*1/*3,*3/*3)	33 17	43.1±27.18 58.29±28.18

Association between genotype and ADP-induced platelet aggregation (U) at *p*-value<0.05.

## Discussion

In this study, we analyzed the pharmacogenetics impact of CYP2C19 polymorphisms on the anti-platelet activity of clopidogrel. The location of CYP2C19 is within a cluster of cytochrome P450 genes on the chromosome 10q24 containing 8 introns and 9 exons.^13^ The CYP2C19*2 has been shown to be a G®A transition at 681 bp in exon 5 of wild-type CYP2C19*1. This variant allele results in an aberrant splice site and it shifts the reading frame of CYP2C19*2 producing an early-stop codon and a truncated protein.^9^ The CYP2C19*3 involves a G®A variant at 636 bp in exon 4, that also creates a premature stop codon and a truncated protein.^10^ Ethnic differences in CYP2C19 variants differences exist in the allele frequencies of CYP2C19. The CYP2C19*2 allele is the most frequent variant in Caucasian, African-American, and Asian populations. However, the allelic frequency is significantly higher in Asian populations (~30%) than in Caucasians (~13%) and African-Americans (~18%). The CYP2C19*3 allele also occurs more frequently in Asian populations (~10%) compared with other racial groups (1%).^14^,^15^ Individuals carrying CYP2C19*2 or *3 as homozygotes (*2/*2, *3/*3) or compound heterozygotes (*2/*3) are defined as poor metabolizers. Different distributions of poor metabolizers have been observed among different racial groups, with much greater prevalence (10-25%) in Asian populations compared to only 2-3% in Caucasians, and 4% in Africans.^16^ In Saudi Arabia, the reported variant allele frequency for CYP2C19*2 in healthy individuals was found to be (0.112).^17^ In our study, the allele frequencies of CYP2C19*2 and CYP2C19*3 were found to be higher in the non-responders group of clopidogrel (0.38, 0.32) compared with the responders group (0.10, 0.12) of clopidogrel cases. Our study showed a significant difference with the genotype and allele frequencies of CYP2C19*2 and *3 variants between responders and non-responders groups of clopidogrel in ischemic stroke patients.

Clopidogrel requires metabolic activation before inhibiting platelet aggregation to overcome the decreased clopidogrel response in patients who are poor metabolizers to CYP2C19 drug metabolizing enzymes and should increase the loading dose from 300 mg to 600 mg^18^ or increase the maintenance dose from 75 mg to 150 mg.^19^ In this study, we evaluated the effects of CYP2C19 genetic polymorphisms on clopidogrel antiplatelet function. Similar to the findings from other investigators in African-American and Caucasian populations.^20^,^21^ Our results have shown that CYP2C19*2 and *3 genotypes modified the effect of clopidogrel. The present study was strengthened by testing the influence of these SNPs on platelet aggregation in parallel as measured by MEA assay.^22^

In our study the ADP induced platelet aggregation (U) level for CYP2C19*2, CYP2C19*3 polymorphism for variant allele was found to be higher compared to the wild type allele in responders, non-responders group of clopidogrel treated ischemic stroke patients and concluded that CYP2C19*2 or *3 LOF alleles were independently associated to ADP-PA measurement.^23^ Hulot and colleagues^24^ showed that the CYP2C19 loss-of-function allele is associated with a marked decrease in platelet responsiveness to clopidogrel in 28 young healthy male subjects treated for 7 days with clopidogrel 75 mg/day. Only CYP2C19*2 and *3 polymorphisms have been demonstrated to be a strong determinant of reduced active clopidogrel metabolite formation corresponding to the studies in Caucasians.^25^,^26^ Concerning the clinical outcome of patients treated with clopidogrel, our results reported here are in agreement with a number of prior studies and confirm the pivotal role of CYP2C19*2 and *3 as genetic markers for platelet aggregation and clopidogrel response.

In conclusion, our findings provide certain evidence on the genetic effect of CYP2C19 on clopidogrel responsiveness in stroke patients from Saudi Arabia, but whether the prognosis data associated with CYP2C19 geno-types can be used to guide individualized and thus optimizing the therapeutic regimens for the secondary prevention of stroke needs further studies. Although our study has a limited sample size, findings of the present study could be of interest considering clopidogrel response variability has been exhaustively studied in ischemic heart disease, but studying it in patients with ischemic stroke represents a field of opportunity. Further studies in a large population are needed to confirm these findings. However, further studies are required to investigate other likely factors involved in clopidogrel resistance, and there is also a requirement for a larger study to better assess the role of genotyping in the evaluation of the phenomenon of clopidogrel resistance.
